# Feasibility of achieving the 2025 WHO global tuberculosis targets in South Africa, China, and India: a combined analysis of 11 mathematical models

**DOI:** 10.1016/S2214-109X(16)30199-1

**Published:** 2016-10-06

**Authors:** Rein M G J Houben, Nicolas A Menzies, Tom Sumner, Grace H Huynh, Nimalan Arinaminpathy, Jeremy D Goldhaber-Fiebert, Hsien-Ho Lin, Chieh-Yin Wu, Sandip Mandal, Surabhi Pandey, Sze-chuan Suen, Eran Bendavid, Andrew S Azman, David W Dowdy, Nicolas Bacaër, Allison S Rhines, Marcus W Feldman, Andreas Handel, Christopher C Whalen, Stewart T Chang, Bradley G Wagner, Philip A Eckhoff, James M Trauer, Justin T Denholm, Emma S McBryde, Ted Cohen, Joshua A Salomon, Carel Pretorius, Marek Lalli, Jeffrey W Eaton, Delia Boccia, Mehran Hosseini, Gabriela B Gomez, Suvanand Sahu, Colleen Daniels, Lucica Ditiu, Daniel P Chin, Lixia Wang, Vineet K Chadha, Kiran Rade, Puneet Dewan, Piotr Hippner, Salome Charalambous, Alison D Grant, Gavin Churchyard, Yogan Pillay, L David Mametja, Michael E Kimerling, Anna Vassall, Richard G White

**Affiliations:** a TB Modelling Group, TB Centre, London School of Hygiene and Tropical Medicine, London, UK; b Department of Global Health and Development, London School of Hygiene and Tropical Medicine, London, UK; c Department of Clinical Research, London School of Hygiene and Tropical Medicine, London, UK; d Faculty of Epidemiology and Public Health, London School of Hygiene and Tropical Medicine, London, UK; e Department of Global Health and Population, Harvard TH Chan School of Public Health, Boston, MA, USA; f Institute for Disease Modeling, Seattle, WA, USA; g Department of Infectious Disease Epidemiology, Imperial College London, London, UK; h Public Health Foundation of India, Delhi NCR, India; i Stanford Health Policy, Centers for Health Policy and Primary Care and Outcomes Research, Stanford University, Stanford, CA, USA; j Management Science and Engineering Dept, Stanford University, Stanford, CA, USA; k Department of Biology, Stanford University, Stanford, CA, USA; l Department of Medicine, Stanford University, Stanford, CA, USA; m Johnson & Johnson Global Public Health, Raritan, NJ, USA; n Institute of Epidemiology and Preventive Medicine, National Taiwan University, Taipei, Taiwan; o Department of Epidemiology, Johns Hopkins Bloomberg School of Public Health, Baltimore, MD, USA; p IRD, UMMISCO, Bondy, France; q Department of Epidemiology and Biostatistics, College of Public Health, University of Georgia, Athens, GA, USA; r The Burnet Institute, Melbourne, Australia; s The Victorian Infectious Diseases Service, at the Peter Doherty Institute, Melbourne, Australia; t Department of Microbiology and Immunology, the University of Melbourne at the Peter Doherty Institute, Melbourne, Australia; u Department of Epidemiology of Microbial Diseases, Yale School of Public Health, New Haven, CT, USA; v Avenir Health, Glastonbury, CT, USA; w Strategic Information Department, The Global Fund, Geneva, Switzerland; x Department of Global Health, University of Amsterdam, Amsterdam, Netherlands; y Amsterdam Institute for Global Health and Development, Academic Medical Center, University of Amsterdam, Amsterdam, Netherlands; z Stop TB Partnership, Geneva, Switzerland; aa Bill and Melinda Gates Foundation, China Office, Beijing, China; ab National Center for Tuberculosis Control and Prevention, Chinese Center for Disease Control and Prevention, Beijing, China; ac Epidemiology and Research Division, National Tuberculosis Institute, Bangalore, India; ad World Health Organization, Country Office for India, New Delhi, India; ae The Bill & Melinda Gates Foundation, New Delhi, India; af Aurum Institute. Johannesburg, South Africa; ag School of Public Health, University of Witwatersrand, Johannesburg, South Africa; ah National Department of Health, Pretoria, South Africa; ai Bill and Melinda Gates foundation, Seattle, WA, USA (currently KNCV Tuberculosisn Foundation, The Hague, Netherlands)

## Abstract

**Background:**

The post-2015 End TB Strategy proposes targets of 50% reduction in tuberculosis incidence and 75% reduction in mortality from tuberculosis by 2025. We aimed to assess whether these targets are feasible in three high-burden countries with contrasting epidemiology and previous programmatic achievements.

**Methods:**

11 independently developed mathematical models of tuberculosis transmission projected the epidemiological impact of currently available tuberculosis interventions for prevention, diagnosis, and treatment in China, India, and South Africa. Models were calibrated with data on tuberculosis incidence and mortality in 2012. Representatives from national tuberculosis programmes and the advocacy community provided distinct country-specific intervention scenarios, which included screening for symptoms, active case finding, and preventive therapy.

**Findings:**

Aggressive scale-up of any single intervention scenario could not achieve the post-2015 End TB Strategy targets in any country. However, the models projected that, in the South Africa national tuberculosis programme scenario, a combination of continuous isoniazid preventive therapy for individuals on antiretroviral therapy, expanded facility-based screening for symptoms of tuberculosis at health centres, and improved tuberculosis care could achieve a 55% reduction in incidence (range 31–62%) and a 72% reduction in mortality (range 64–82%) compared with 2015 levels. For India, and particularly for China, full scale-up of all interventions in tuberculosis-programme performance fell short of the 2025 targets, despite preventing a cumulative 3·4 million cases. The advocacy scenarios illustrated the high impact of detecting and treating latent tuberculosis.

**Interpretation:**

Major reductions in tuberculosis burden seem possible with current interventions. However, additional interventions, adapted to country-specific tuberculosis epidemiology and health systems, are needed to reach the post-2015 End TB Strategy targets at country level.

**Funding:**

Bill and Melinda Gates Foundation

## Introduction

In May, 2014, the World Health Assembly approved the post-2015 End TB Strategy, setting “ambitious but feasible” targets for reducing the global burden of tuberculosis by 2035.^1^, ^2^ The strategy is aiming for a 50% reduction in global tuberculosis incidence and a 75% reduction in global tuberculosis mortality by 2025, and 90% and 95% reductions in these outcomes, respectively, by 2035.^2^ Policy makers must identify what interventions, and at which level of scale-up, will be needed to meet these targets at country level.

The End TB targets are deliberately ambitious, and any single intervention (defined here as a group of activities leading to an improvement in a specific area of tuberculosis control—eg, treatment outcomes) is unlikely to achieve these goals.^3^ Instead, national tuberculosis programmes will need improvements across the tuberculosis care pathway, together with preventive measures.

The End TB Strategy describes two phases of future efforts to control tuberculosis.^2^ In phase 1, the focus of this Article, progression towards the 2025 milestones will largely depend on optimising the use of existing tools, enabled by investments in universal health coverage and social protection.^4^ Post-2025 in phase 2, novel tools (diagnostics, drugs, and vaccines) are expected to enable further acceleration of tuberculosis decline towards the 2035 targets.^1^ For both phases of the End TB Strategy, policy makers require guidance about which interventions and technologies to use—questions that are unlikely to be answered by empirical studies, given the difficulty of testing all possible approaches at high scale before policy decisions are made.

**Research in context**

**Evidence before this study**

The post-2015 Global TB Strategy envisions and is aiming for a 50% reduction in tuberculosis incidence and a 75% reduction in tuberculosis mortality by 2025, using existing or near-existing tools. Given that this period starts in 2016, there is an urgent need to inform policy discussions on how these targets can be reached on a country level. Modelling can be a powerful tool to address this need by projecting the potential effect of a combination of different interventions. Additionally, by using multiple models to address the same question, it can be used to identify findings robust to between-model variation, increasing confidence in the conclusions. We reviewed existing modelling studies that assessed the individual and combined impact of a range of existing interventions, and other multimodelling exercises in the field of tuberculosis. We built on a systematic review by the TB Modelling and Analysis Consortium who gathered all tuberculosis modelling papers and extended the review to June, 2015. PubMed was searched using the following search query: (tuberculosis OR TB) AND ((mathem* AND (model OR models)) OR (mathem* modell*) OR (mathem* modeling) OR (modeling OR modelling) OR “Population Dynamics” [MeSH Terms] OR “Population Dynamics” OR “System Dynamics” OR “Computer Simulation” OR “Computer Simulation” [MeSH Terms])”. We also did specific searches in mathematical modelling journals, and searched private libraries, and references of existing modelling reviews. We only included English language papers and we used no date restrictions.

Ours is the first study to compare multiple tuberculosis models to answer a public health question. Single models have usually evaluated a single intervention, making it difficult to understand the full potential, including potential synergy, or non-synergy, of a combination of interventions implemented simultaneously. Other multimodel exercises have been published, most notably in the field of HIV, which focused on questions around antiretroviral therapy scale-up to inform UNAIDS policy.

**Added value of this study**

This study highlights the uncertainty in the natural history of tuberculosis that drives between-model differences, while still identifying relatively consistent findings of public health importance. It explores how a range of existing interventions across the tuberculosis care pathway, scaled up to country-specific levels, can take China, India, and South Africa towards the 2025 global tuberculosis targets. Our results show that expansion of existing interventions should enable South Africa to reach the 2025 targets, while for India and China additional context-specific activities are likely to be needed.

**Implications of all the available evidence**

Although major reductions in tuberculosis burden seem possible with current tools and 2025 targets might be met in South Africa, additional interventions, adapted to the country-specific tuberculosis epidemiology and health systems, are likely to be needed to reach the post-2015 Global TB Targets in other key countries such as China and India. These might include interventions such as tackling the latent tuberculosis infection reservoir in elderly people in China and undernutrition in India. This decision making can be informed by rigorous data analysis and the logical framework that mathematical models provide.

Mathematical modelling is a powerful tool to support policy discussions, because several hypothetical intervention strategies can be compared in a systematic framework to project future trends.^5^, ^6^, ^7^ Multimodel exercises for HIV^8^, ^9^ have illustrated how differences in model design can influence results, yet this structural variation is not apparent in a single-model analysis. By comparing answers to the same question using different models, we can identify findings robust enough to account for between-model variation, and as such contribute to the evidence needed to commit the resources for national-level policy initiatives. The points where model projections diverge can signal important knowledge gaps to be addressed by future research. Paired with information on use of resources, projections of health impact also can be used to estimate the cost-effectiveness of competing policy options and to design optimum policy portfolios, which is beyond the scope of this paper.

To provide results relevant to individual countries, modelled analyses should be consistent with existing evidence on local tuberculosis epidemiology (eg, incidence, mortality, prevalence, and multidrug-resistant [MDR] tuberculosis) and tuberculosis control activities (eg, treatment success, and linkage to care), and tailor intervention scenarios to local needs and capabilities. Because the required information is not always available in a systematic way in the public domain, involvement of local experts is key.

In this Article, we describe epidemiological projections from 11 independently developed dynamic transmission models of tuberculosis, exploring the feasibility of the 2025 End TB Strategy targets in China, India, and South Africa. These analyses explore a range of policy scenarios, incorporating perspectives from national tuberculosis programmes and advocacy communities. China, India, and South Africa account for approximately 40% of the global tuberculosis burden^10^ and are ideal to explore the feasibility of the targets in a country context because of their distinct combinations of epidemiological characteristics, health systems, and levels of tuberculosis prevention and care pathway activities. The likelihood of achieving the global targets will depend, to a large extent, on progress in these high-burden countries.

## Methods

### Participating models

After a global call from the TB Modelling and Analysis Consortium for expressions of interest, 11 modelling groups contributed results for at least one of the countries (China, India, or South Africa). These models varied in their frameworks, population stratifications, and approaches used to model disease and intervention mechanisms. An overview of participating models and references is in table 1.

**Table 1:** Description of mathematical models

	**Country**	**Model type**	**Model calibration**	**Age structure**	**Sex strata**	**Population strata**	**Results reported**	Interventions modelled*
NTU^31^	China	D	Manual	15+ years	No	MDR, health-care sector, treatment history	Single	All
ICPHFI^32^	India	D	Algorithmic	15+ years	No	MDR, treatment history, HIV (2 strata), health-care sector	Single	All
STAMP^33^	India	I	Grid Search	1-month age groups	Yes	MDR, treatment history, health-care sector, time since infection and activation	Stoch	All
Hopkins^34^	South Africa	D	Manual	Single age group (15+ years)	No	MDR, health-care sector, treatment history, HIV/ART/CD4 status (5 strata)	Single	All
IRD^35^	South Africa	D	Manual	1-month age groups	Yes (HIV only)	HIV/ART/CD4 status (5 strata)	Single	IPT for ART
SIPTM	South Africa	D	Manual	<15, 15-19, 19< years	No	HIV/ART/CD4 status (5 strata)	Single	IPT for ART
UGA^36^	South Africa	D	Manual	<15 and 15+ years	No	MDR, health-care sector, HIV/ART/CD4 (3 strata)	Single	All
IDM^21^	South Africa, China	I	South Africa: Manual calibration China: Bayesian (incremental mixture importance sampling);	Explicit age	No	MDR, health-care sector, treatment history, HIV/ART/CD4	Stoch	All
Harvard^7^	South Africa, India, China	D	Bayesian	Single age group	No	MDR, health-care sector, treatment history, HIV/ART/CD4 (9 strata)	Single	All
AuTuMN^37^	South Africa, India, China	D	Algorithmic	<15 and 15+ years	No	MDR, health-care sector, South Africa: HIV/ART/CD4 (5 strata)	Single	All
TIME^38^	South Africa, India, China	D	Manual	<15 and 15+ years	No	MDR, treatment history HIV/ART/CD4 status (11 strata)	Single	All

D=deterministic compartmental model. I=individual-based, stochastic model. Sex strata: Yes=natural history or care pathway parameters different for male and female patients; No=no sex stratification in models. MDR=multidrug-resistant. ART=antiretroviral therapy. Single=single parameter set. Stoch=average (mean or median) of stochastic simulations.

* See table 2 for details of interventions.

### Data and country context for baseline

To calibrate the models and provide a baseline scenario, modellers were provided calibration targets reflecting tuberculosis burden (as incidence and mortality) in 2012^11^, ^12^ and tuberculosis control activities. Because half the participating models only included adult groups (aged ≥15 years), calibration targets and results also focused on adults (appendix). For China, the calibration targets included 2000 and 2010 tuberculosis prevalence values as estimated by national surveys.^13^ For South Africa, models were calibrated to reflect an estimated 2–5% decline in the annual incidence of tuberculosis in 2012 and projected scale-up of antiretroviral therapy (ART) coverage to 77% of HIV-positive adults by 2025^10^, ^14^ (see appendix section 1 for additional details of the calibration process, including sources).

### Intervention scenarios

**Figure 1:**
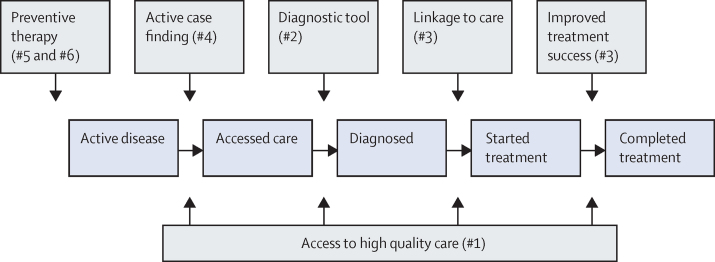
TB Care and Prevention framework The patient care pathway from disease to completion of treatment (blue boxes and arrows). Areas affected for enhancing current tuberculosis programme activities (ie, intervention scenarios) are shown in grey boxes and arrows, with the number (#x) to link them to activities in table 2 and the appendix section 3.

We defined a framework of enhancements of tuberculosis-programme activities using existing tools, which were grouped into intervention scenarios (figure 1). Through detailed discussions with representatives from national tuberculosis programmes and the global advocacy community (Stop TB Partnership), we defined two distinct levels of scale-up within those intervention scenarios. Table 2 summarises national tuberculosis programme and advocacy scenario sets for each country; see appendix seciton 2 for members and affiliations for each group.

**Table 2:** Summary of modelled intervention scenarios and target values for China, India, and South Africa

	**China**			**India**			**South Africa**		
	Activities*	Base value	Target value†	Activities*	Base value	Target value	Activities*	Base value	Target value
**#1. Increase access to high quality care**‡									
Reduce proportion not accessing any tuberculosis care	Government subsidises tuberculosis care, and compensates patients for incurred costs	5%	3·75%(NTP), 0% (A)	Government subsidises diagnostic and treatment costs in private sector, expanding number of clinics and opening times of tuberculosis care	9·5%	4·75% (NTP), 0% (A)	Improve geographical access through outreach clinics	5%	0% (NTP), 0% (A)
Of those with care access, increase proportion accessing high quality care	Same technology and approaches available in hospital and CDC sector	80%	95% (NTP), 100% (A)	Government subsidises use of high quality tools and protocols in private sector	50%	90% (NTP), 100% (A)	Tuberculosis symptom screening for all health clinic attendees to ensure all in need receive tuberculosis diagnosis	20%	100% (NTP), 100% (A)
**#2. Diagnosis of disease and MDR**§									
Replace smear microscopy with molecular diagnostic (eg, GeneXpert) as first-line test	Replacement of smear microscopy with molecular diagnostic in facilities	0%	100% (NTP), 100% (A)	Replacement of smear microscopy with molecular diagnostic in facilities	0%	30% (NTP), 100% (A)	Not modelled because rollout of GeneXpert has been implemented already	100%	Not modelled
**#3. Improve post-diagnosis care**§									
Reduce pretreatment loss to follow-up: first-line	Compensation for patient costs	3%	1·5% (NTP), 0% (A)	Provide patient incentives for treatment initiation	10%	5% (NTP), 0% (A)	Expand monitoring and assessment capacity, implement mhealth and outreach teams to trace patients in communities	17%	5% (NTP), 0% (A)
Reduce pretreatment loss to follow-up: MDR	Compensation of patient costs, improvements in speed of diagnosis and referral	50%	15% (NTP), 0% (A)	Linkage to social welfare programmes, including nutritional support	11%	5% (NTP), 0% (A)	As above	50%	15% (NTP), 0% (A)
Increase first-line treatment success	Implement patient support strategies including health and case management	82%	90% (NTP), 95% (A)	Provide incentives and linkage to welfare programmes	75%	85% (NTP), 90% (A)	Provide patient with adherence counselling and psychosocial support, as well as improved monitoring and evaluation	76%	85% (NTP), 85% (A)
Increase MDR treatment success	Improve patient monitoring (mhealth) and side-effect amelioration	35%	65% (NTP), 80% (A)	As above	48%	67% (NTP), 80% (A)	All of above, as well as decentralisation of electronic register	50%	67% (NTP), 75% (A)
**#4. Active case finding in general population**									
Periodically screen a proportion of the general population for tuberculosis disease	As general description	0%	0% (NTP), 30% (A)	As general description	0%	1·6% (NTP), 30% (A)	As general description	0%	0% (NTP), 50% (A)
**#5. Active case finding followed by treatment of latent tuberculosis**									
Provide LTBI screening and preventive therapy when positive to proportion of active case finding population where active tuberculosis was excluded	As general description	0%	0% (NTP), 100% (A)	As general description	0%	0% (NTP), 100% (A)	As general description	0%	0% (NTP), 100% (A)
**#6. Continuous IPT for ART-receiving population**									
Provide continuous IPT as part of ART in PLWHIV.	Not modelled	..	..	Not modelled	..	..	Includes preinitiation screening, and rescreening of those lost to follow-up	5%	80% (NTP), 100% (A)
**#7. Combination**									
Scale up all interventions simultaneously	All of above	..	..	All of above	..	..	All of above	..	..

Information describes the general intervention effects to be modelled, which were adapted to fit within specific model structures (see appendix section 3 for details). Target value=absolute value. NTP=national tuberculosis programme scenario. A=advocacy scenario. PLWHIV=people living with HIV. CDC=Centers for Disease Control. mhealth=mobile health. MDR=multidrug resistant. LTBI=latent tuberculosis infection. IPT=isoniazid preventive treatment. ART=antretroviral therapy.

* Summarises the activities proposed by the NTP scenario-setters to enhance current programme performance.

† Scale-up to target value started in 2016 and usually reached in 2020.

‡ High quality care describes the best performing sector of all tuberculosis care providers—eg, public sector in India, CDC sector in China.

§ Intervention scenarios for diagnosis (#2) and care (#3) apply to population accessing high quality care only.

The first intervention scenario looked at improvements in access to high-quality care, defined as individuals with tuberculosis disease having access to the best level of care available in their local context. Tuberculosis care in China and India is provided through many health-care providers.^15^ Since the quality of care is known to differ strongly between providers,^16^, ^17^ the intervention scenarios explored what could be achieved if a higher proportion of the population of patients with tuberculosis disease received the higher quality care as provided in the Centers for Disease Control (China) or the public sector (India). For South Africa, models explored a new policy of expanded screening of health-centre visitors for symptoms of tuberculosis disease.

A second set of intervention scenarios modelled improvements in the tuberculosis care pathway, which included replacing sputum smears with a molecular diagnostic test such as GeneXpert as the first laboratory test, increasing linkage to care for individuals diagnosed with tuberculosis disease, and improved treatment outcomes for those linked to care. We also estimated the effect of active case finding for tuberculosis disease in the general population, implemented as simple screening of a proportion of the population for disease (table 2, appendix), either on its own, or as screening for active disease along with preventive therapy for individuals with latent tuberculosis infection. For South Africa, an additional intervention scenario estimated the impact of providing continuous isoniazid preventive therapy, with screening for active disease before initiation for individuals receiving ART. A combination intervention scenario estimated the overall impact of all interventions run simultaneously. Only models that contributed results to all individual interventions reported the combined intervention.

Under the advocacy scenarios, models estimated the potential effect on tuberculosis incidence and mortality if countries were able to screen and treat 30% (India and China) or 50% (South Africa) of the general population (as a proxy for identifying a similar proportion of the burden through active-case finding in high-risk groups) for tuberculosis disease and latent tuberculosis infection twice a year (table 2). With the exception of the active case finding and preventive therapy for the general population, each intervention scenario was operationalised as specific programmatic activities tailored to each country context (table 2). Modellers were asked to reflect these activities as closely as possible using their respective model structure and parameterisation (see appendix section 3 for guidance provided and the implementation of the intervention scenarios for each model).

Economic development and related investments in universal health coverage, components of Pillar 2 in the End TB Strategy^1^ were considered critical enablers of other intervention scenarios—eg, for access to high-quality care and treatment success (table 2, appendix section 3). Thus, we did not model separately potential benefits for interventions such as achievement of universal health coverage, cash-transfer programmes, or the preventive effect of poverty-reduction efforts on tuberculosis outcomes.

Models reported on tuberculosis incidence, mortality, and prevalence for the period 1990–2025. Because half the models captured adults only (table 1), our main outcomes were the change in adult (aged ≥15 years) incidence and mortality between 2015 and 2025 in the baseline scenarios. Additionally, we recorded the incremental impact of individual intervention scenarios and the overall impact of the baseline plus the combination intervention scenarios, and the cumulative cases and deaths averted. Additional outcomes reported by modellers included MDR tuberculosis prevalence in new or retreatment cases, latent tuberculosis infection prevalence, and the proportion of disease after recent infection. These outcomes were used to understand the differences between models and ensure internal model consistency. After fitting to the calibration targets, modelling groups were provided with guidance on how to implement the intervention scenarios (appendix section 3) in view of the differences in model structures. Additionally, we established minimum requirements to model structure for contribution to each scenario (appendix section 3).

### Role of the funding source

The funder of the study had no role in study design, data collection, data analysis, data interpretation, or writing of the report. The corresponding author had full access to all the data in the study and had final responsibility for the decision to submit for publication.

## Results

The modelling took place between March and Dec, 2014. Of 11 participating models, six provided projections for China, five for India, and eight for South Africa. Three modelling groups (Harvard, AuTuMN and TIME) contributed results for all three countries and one model (IDM) modelled the China and South Africa epidemics. Of 11 models, nine provided results for all interventions (table 1).

**Figure 2:**
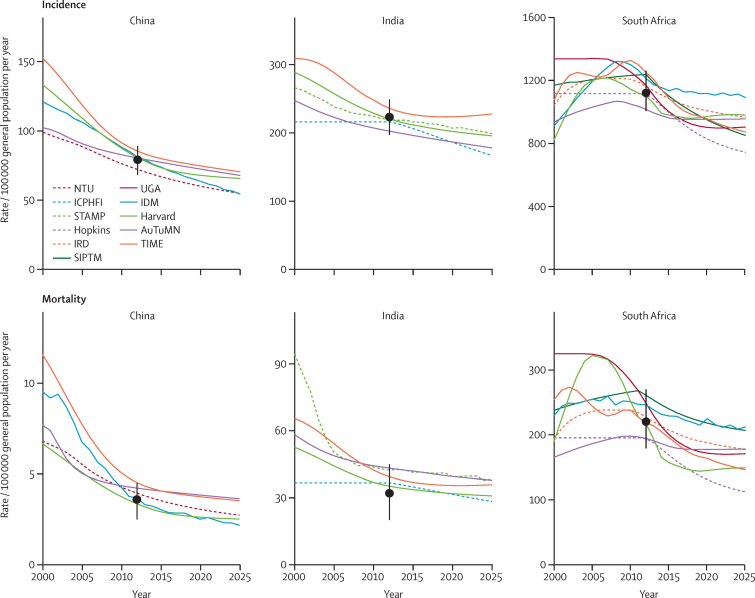
Baseline calibration and projections for China, India, and South Africa Y-axes scales have different values. Coloured lines show model results, black dots and lines show required calibration ranges. Additional calibration targets included prevalence surveys (China, 2000 and 2010) and 2–5% annual decline in incidence (South Africa). See appendix section 1 and 4 for details.

Figure 2 shows baseline calibration and projections for incidence and mortality. In China, where models were calibrated to 2000 and 2010 prevalence targets (appendix sections 1 and 4), historical and projected trends were similar between models. For India, the historical uncertainty was propagated in the projections, although most models predicted a declining incidence trend consistent with recent WHO projections.^10^ In South Africa, models were calibrated to an epidemiological burden and trend in 2012, but diverged over time as the projected baseline change in incidence between 2015 and 2025 ranged from 0 to 25%. For China and India, baseline changes in incidence were 11–27% and 0–19%, respectively, suggesting that with tuberculosis-programme activity, the tuberculosis burden was most likely to continue its decline.

**Figure 3:**
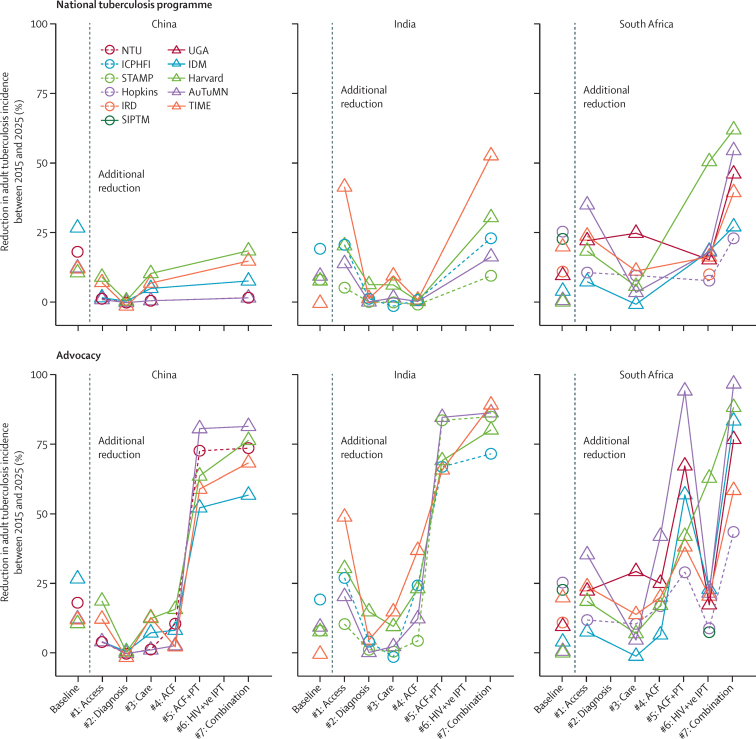
Impact of interventions on incidence for national tuberculosis programmes and advocacy scenarios Figure shows the impact of baseline (left of dotted line) and incremental (excluding baseline, right of dotted line) impact of individual intervention scenarios (triangles and circles). Lines between models are for illustration of within-model impact of interventions. Models had to reflect the activities as provided by scenario setters (see table 2) as best as possible within their model framework, and provide an implementation narrative (see appendix section 3). Inevitably, simplification will have occurred to fit the intervention within the model structure. For example, in South Africa, the method of implementing the intervention scenario of isoniazid preventive therapy for HIV positive individuals receiving antiretroviral therapy will depend on whether the model had a separate compartment for isoniazid preventive therapy to track the number of individuals who were screened (as part of annual re-screening for tuberculosis) and have separate tuberculosis progression rates. See appendix section 3 for guidance and specific implementation.

In the national tuberculosis programme scenarios, the incremental contribution of individual intervention scenarios (compared with the baseline) differed strongly between countries. In China, the additional impact of individual interventions on tuberculosis incidence and mortality was small for all scenarios (figure 3, appendix section 4). By contrast, for India, improving access to high-quality care substantially reduced tuberculosis incidence beyond baseline trends by a median of 20% (range 5–41%). In the same setting, activities solely aimed at further improving care for patients already accessing high-quality care made little difference to baseline trends. In South Africa, although we noted some between-model variation, most intervention scenarios showed substantial impact, with prevention (#6: continuous isoniazid preventive therapy for individuals receiving ART), case finding (#1: screening at primary-health clinics), and improvements in linkage to care and treatment success (#3) reducing tuberculosis incidence by a further median of 16% (range 8–51%), 20% (7–35%), and 8% (0–25%), respectively.

**Figure 4:**
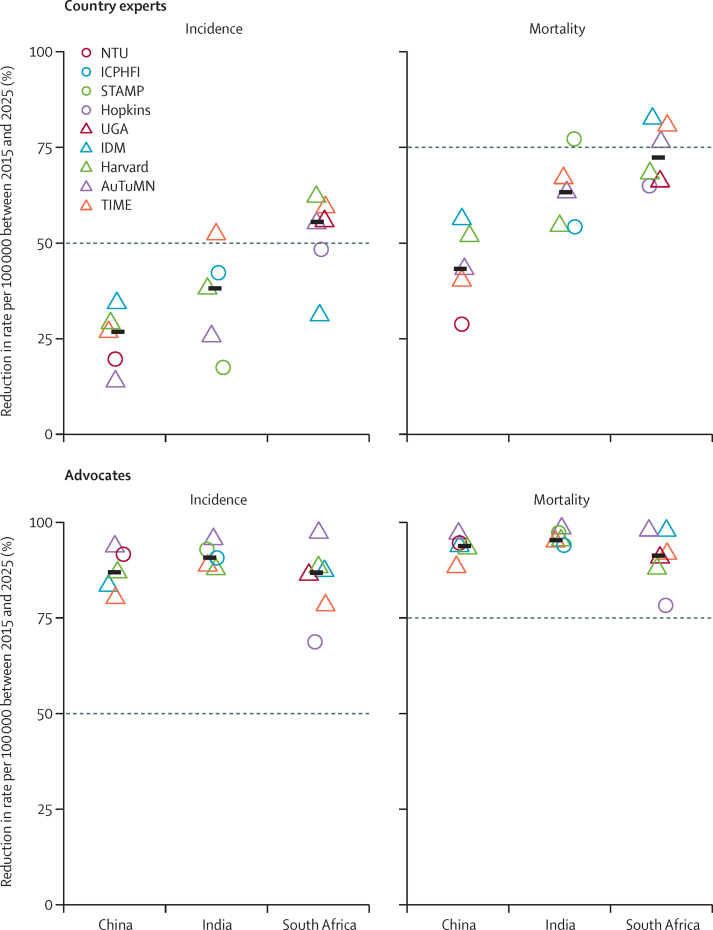
Combination intervention impact on incidence and mortality in scnearios for national tuberculosis programmes (top row) and advocacy (bottom row) Figure shows individual model impact (triangles and circles) and median impact (black bars). Dotted lines show 2025 milestones of 50% reduction in incidence (left column) and 75% reduction in mortality (right column).

In South Africa, the 2025 End TB Strategy targets seem feasible, because the model projections showed that a combination of prevention, case finding, and improvements in care (figure 4) reduced incidence and mortality with a median of 55% (range 31–62%) and 72% (65–82%), respectively, and averted a cumulative 1·2 million (0·7 million–1·8 million) cases of tuberculosis and 298 000 (193 000–453 000) deaths from tuberculosis between 2015 and 2025. In China, median cases and deaths averted by the combination intervention scenarios were 312 000 (range 42 000–764 000) cases of tuberculosis and 65 000 (10 000–93 000) deaths from tuberculosis. In India the corresponding figures were 3·1 million (1·2 million–5·8 million) cases of tuberculosis and 1·1 million (0·8 million–2·1 million) deaths from tuberculosis. Despite these projected substantial health gains, the proportional reductions between 2015 and 2025 were estimated to fall short of the post-2015 End TB Strategy targets for China and India (figure 4).

The impact of annual screening for active disease (#4 active-case finding in the general population) was small when compared with the effect of treating latent tuberculosis infection, particularly for China where #5 (active-case finding followed by treatment of latent tuberculosis infection) achieved a median of 64% (range 52–81%) reduction in tuberculosis incidence (figure 3), and the impact of the combined intervention exceeded the 2025 global targets.

## Discussion

Using multiple, independently developed tuberculosis transmission models, we explored the feasibility of achieving the post-2015 End TB Strategy targets in three high-burden countries, each with different epidemiology and existing levels of tuberculosis control. By projecting the impact of combinations of existing tools in China, India, and South Africa, we showed the importance of country context in assessing whether and how these global targets might be achieved at a country level. For South Africa, the 2025 milestones of 50% reduction in incidence and 75% reduction in mortality appear feasible with existing tools, whereas for India and China, these targets appear unfeasible.

Contrasting results between countries reflect the differences between epidemiological context and responses of local tuberculosis epidemics. Whereas in China, two decades of steady improvement in reach and quality of basic tuberculosis services have led to nearly a two-thirds reduction in tuberculosis prevalence between 1990 and 2010,^13^ South Africa's tuberculosis programme was overwhelmed by the effects of HIV,^18^ and is only just turning a corner.^10^, ^19^ The room for further improvement with current tools differs widely between countries, which leaves high-performing tuberculosis programmes, such as the one in China, with the question of how to achieve additional reductions. India faces specific challenges around private providers of tuberculosis care, who are common throughout the southeast Asia region, and our results show that improving the quality of tuberculosis care in the private sector is essential. This process is underway, partly through an expansion of government subsidies to pay for individuals to access effective tuberculosis diagnosis and treatment through the private sector.^20^

The post-2015 End TB Strategy targets are laudable in their ambition, and describe what would be a great achievement in tuberculosis control, but our results show that the targets and the tools enlisted to achieve them will need adapting to provide countries with a path that is both ambitious but also feasible. Achievement of the targets at a global level will be challenging, because more modest contributions from one country will need to be compensated by other countries going beyond these already highly ambitious targets. Additionally, for countries where the standard package of interventions is likely to be insufficient, new strategies need to be developed that tackle country-specific drivers, such as the ageing population of individuals with tuberculosis in China^21^ and high levels of undernutrition in India.^22^, ^23^

As they stand, the advocacy scenarios would involve community-based tuberculosis screening for substantial parts of the population, twice a year. With current tools, this raises substantial issues around feasibility, resources, and evidence for impact.^24^ Also, we did not quantify potential negative effects, including false-positive treatment, and regimen side-effects. However, the advocacy scenarios illustrate a key point of addressing the latent tuberculosis infection reservoir in the population, particularly for settings like China where current high levels of tuberculosis programme performance (and resulting relatively minor contribution of transmission to tuberculosis incidence^25^) means substantial gains can be made in this area. Tools are needed to reduce the volume (and associated costs) of screening for active disease and treatment of latent tuberculosis infection, such as a postexposure vaccine, or a screening test that detects individuals with latent tuberculosis infection who are likely to progress in the next 5–10 years,^26^ all of which are part of Pillar 3 of the End TB strategy.^1^

We collated the best available data from published reports and country experts, but emphasise that improved information about tuberculosis epidemiology and the current tuberculosis care pathways is still needed. For example, substantial uncertainty exists about tuberculosis incidence and mortality in India, prevalence of latent tuberculosis infection, and treatment volume provided in public and private sectors, which can affect model projections.^10^, ^15^ The epidemiological effect of activities often relied on expert opinion in the absence of reliable data, which adds uncertainty to model projections. Further evidence about efficacy of interventions activities is needed to inform policy discussions at a global and national level.

In this project we aimed to examine the impact of major policy options in the tuberculosis response. As such, some specific interventions that might be considered for specific settings were not included such as addressing undernutrition in India and age-specific screening for latent tuberculosis infection in China, which have been explored in other models. 21, 22, 23 Also, we did not include scenarios that focused on active case finding in high-risk groups, such as miners or people living in informal settlements. To adequately capture these dynamics, and the impact of targeted interventions in these populations in a reasonable way, we believe that epidemiological models require specific model structure, and credible data on size and tuberculosis burden in each population, as well as a reasonable estimate of mixing within and between the general and high-risk populations. When more data become available, these choices can be revisited.

We did not report parametric uncertainty, or the relationship between model structure and predicted outcomes. These issues represent areas for future research. What is clear is that, as models aim to capture greater complexity, the structural and parametric uncertainty that can be expressed increases. One illustration is the more pronounced divergence in baseline model projections for South Africa where HIV is a key driver of the tuberculosis burden. Models that capture the interaction between tuberculosis and HIV and the effect of ART create additional opportunities for model differences and resulting divergence of baseline projections. However, since there is no one true model structure, multimodel exercises such as ours are important to identify findings robust to the structural uncertainty, as we have here.

Our study provides unique insights on the feasibility of these global epidemiological 2025 targets at the country level, and illustrates the challenges ahead. In further work, these epidemiological projections have been linked to costs^39^ to explore cost-effectiveness, affordability, and poverty alleviation. Such information is vital as policy makers and the global tuberculosis community assess the health gains and economic costs that would come with scaling-up existing tools to meet the first targets of the End TB Strategy.
