# Supplementary material for: Feasibility of achieving the 2025 WHO global tuberculosis targets in South Africa, China, and India: a combined analysis of 11 mathematical models
Source: Lancet Glob Health. 2016 Oct 6;4(11):e806–15. doi: 10.1016/S2214-109X(16)30199-1 (PMC6375908; doi:10.1016/S2214-109X(16)30199-1)

THE LANCET Global Health

Supplementary appendix

This appendix formed part of the original submission and has been peer reviewed. We post it as supplied by the authors.

Supplement to: Houben RMGJ, Menzies NA, Sumner T, et al. Feasibility of achieving the 2025 WHO global tuberculosis targets in South Africa, China, and India: a combined analysis of 11 mathematical models. *Lancet Glob Health* 2016; published online Oct 6. http://dx.doi.org/10.1016/S2214-109X(16)30199-1.

Feasibility of achieving the 2025 WHO global tuberculosis targets in South Africa, China, and India: a combined analysis of 11 mathematical models

Houben et al.

Paper appendix

This document provides supplementary information to the main paper. It has four sections:

- Section 1 (pg 1- 5): Information used to calibrate the models to the TB epidemiology of China, India and South Africa.
- Section 2 (pg 6): List of country experts and advocates who contributed to setting the country scenarios.
- Section 3 (pg 7-24): Information used to model the intervention scenarios for each country
- Section 4 (pg 25-27): Additional figures

Section 1: Model Calibration targets

A. BACKGROUND

This document describes the required and optional calibration targets provided to participating modelling groups to calibrate their country models.

Baseline values for model parameters affected by the intervention scenarios (e.g. initial default, treatment success) are summarised in ANNEX 3. Models were required to reflect these baseline values in their models.

B. STRUCTURE OF SECTION

The information is structured by country. Table 1 holds point values and accepted range (and source) for **required** calibration targets. A 'required' target range was only considered to be hit when the central (mean, median, etc..) value of the model results lies on or within the range provided.

Table 2 describes **optional** strong data points (+ range and source) that models should aim to approximate, although they would not lead to model exclusion.

C. AGE STRUCTURE OF INDICATORS AND OUTPUT

As half of participating models only considered adult (>= 15 years old) age groups, adult specific calibration targets were provided. This required an adjustment of values available in the literature, which are usually reported for all age groups.

We used estimates of the proportion of all incident TB cases or TB deaths that occurs in children to calculate what the adult only rate of incidence or mortality is (using UN population estimates as the revised denominator).

For incident TB cases, we used regional estimates from a recent publication (Jenkins et al. Lancet 2014). These proportions were 11.2% for Western Pacific Region (China), 11.7 for South East Asia region (India) and 12.2 for the African region (South Africa).

For mortality the existing official estimates (GTB and IHME) are data (e.g. verbal autopsy) based. Verbal autopsies are not highly accurate for TB, and even more so in children given the difficulty in diagnosing

childhood TB. Also, the data based estimates for mortality were not linked in any way to the proportion of all TB burden found in children, which can cause problems. Another alternative was to apply the Case fatality ratio in notified childhood TB cases to the total burden, but these data are not available.

Given these limitations, we applied a simply but clear approach, assuming that the proportion all TB deaths in children was equal to the proportion of all TB that is in children, i.e. 11.2% for China, 11.7 for India and 12.2 for South Africa. This could reflect a lower overall mortality in children, which would be counterbalanced by the lower detection rate. While this method was clearly imperfect, in the absence of good alternative data and approaches, a clear method was preferable. The resulting mortality rates are listed in the tables.

D. OTHER NOTES

When a year is specified, the target should be hit in the mid-point of that year. For each calibration target a range of values (upper and lower limit) was considered to reflect the existing uncertainty around the point estimate. Models were allowed to hit any value within that range. The source column provides information on where the estimates came from.

For mortality in China we used the widest range of values possible from the GTB and IHME estimates.

CHINA

Table C1. Required calibration targets

Target	Time point	value	lower limit	upper limit	Source
TB disease incidence (n/100k/year) (**adult only**, all types)	2012	79	68	89	*Estimate 11.2% (8.8,13.5) of TB cases occur in children in WPR region*
TB mortality (n/100k/year) (all TB types, **adults only**, all sectors)	2012	3.6	2.5	4.5	*Estimate 11.2% (8.8,13.5) of TB deaths occur in children in China*
Total population size (n in thousands) (**adults only**)	2012	1126659			*Estimate 18% of population is <15* UN pop division. Interpolation of 15+ values between 2010 and 2015
TB disease incidence (n/100k/year) (**all ages**, all types)	2012	73	64	82	2013 GTB report
TB mortality (n/100k/year) (all TB types, **all ages**, all sectors)	2012	3.3	2.4	4.0	IHME GBD data used. HIV negatives only.
Total population size (n in thousands) (**all ages**)	2012	1377065			UN pop division 2012 revision
TB Prevalence (n/100k) (>15y/o, smear positive)	2000	137	123	153	China NTP
TB Prevalence (n/100k) (>15y/o, smear positive)	2010	59	49	72	China NTP

Table C2. Additional strong data points

Target	Time point	value	lower limit	upper limit	Source
TB Prevalence (n/100k) (smear positive, >15y/o)	1990	170	166	174	Prevalence survey
% MDR in new cases	2007	5.7%	4.5%	7.0%	MDR survey
% MDR in retreatment cases	2007	25.6%	21.5%	29.8%	MDR survey

INDIA

Table I1. Required calibration targets

Target	Time point	value	lower limit	upper limit	Source
TB disease incidence (n/100k/year) (**adult only**, all types)	2012	223	197	249	*Estimate 11.7% (9.5,13.0) of TB cases occur in children in SEA region*
TB mortality (n/100k/year) (all TB types, **adults only**, all sectors)	2012	32	20	45	*Estimate 11.7% (9.5,13.0) of TB deaths occur in children in India. Note include HIV positive TB deaths, which will be a small number.*
Total population size (n in thousands) (**adults only**)	2012	872546			*Estimate 29% of population is <15* UN pop division. Interpolation of 15+ values between 2010 and 2015
TB disease incidence (n/100k/year) (**all ages**, all types)	2012	176	159	193	2013 GTB report
TB mortality (n/100k/year) (all TB types, **all ages**, all sectors)	2012	25	17	35	2013 GTB report (including HIV positive deaths)
Total population size (n in thousands) (**all ages**)	2012	1241492			UN pop division 2012 revision

Table I2. Additional strong data points

Target	Time point	value	lower limit	upper limit	Source
Total population size (n in thousands) (**all ages**)	2025	1418744			UN pop division projection

SOUTH AFRICA

Table S1. Required calibration targets

Target	Time point	value	lower limit	upper limit	Source
TB disease incidence (n/100k/year) (**adult only**, all types)	2012	1117	1002	1259	*Estimate 12.2% (10,14.7) of TB cases occur in children in AFR region*
TB mortality (n/100k/year) (all TB types, **adults only**, all sectors)	2012	220	179	270	*Estimate 12.2% (10,14.7) of TB deaths occur in children in South Africa*
Total population size (n in thousands) (**adults only**)	2012	36824			*Estimate 29% of population is <15* UN pop division. Interpolation of 15+ values between 2010 and 2015
TB disease incidence (n/100k/year) (**all ages**, all types)	2012	900	832	990	2014 GTB report
TB mortality (n/100k/year) (all TB types, **all ages**, all sectors)	2012	179	149	212	2014 GTB report
Total population size (n in thousands) (**all ages**)	2012	52386	52230	52541	UN pop division 2012 revision
Annual decline in TB incidence (all ages or adults only)	2012	3%	2%	5%	2014 GTB report

Table S2. Additional strong data points

Target	Time point	value	lower limit	upper limit	Source
HIV prevalence in TB cases (% HIV positive of notified TB cases)	2012	63	57	69	2013 GTB data. No estimates of range so assume +/- 10%
HIV prevalence in adult population (% HIV positive in >=15yo population)	2012	15	14	16	UNAIDS Report on the Global AIDS Epidemic - 2013 and UN pop division 2012 revision
ART coverage in general population (% receiving ART of HIV positive population (15-49 yo))	2012	35	34	37	Global Update on HIV treatment. Estimated from reported numbers of adults on ART and estimated numbers of adults living with HIV. Coverage among eligible population (dependent on CD4 count threshold) is estimated to be 83% [79-87%]

					2013: WHO report in partnership with UNICEF and UNAIDS
% of all TB deaths which are HIV positive	2012	74	54	95	2013 GTB data
Total population size (n in thousands) (**all ages**)	2025	56666	54108	59224	UN pop division projection (medium, low and high fertility projections)

ART in South Africa (2014 UNAIDS estimates)

	% of total HIV positive population to receive ART		
Year	Point estimate	lower bound	Upper bound
2000	0	0	0
2001	0	0	0
2002	0	0	0
2003	0	0	0
2004	1	1	1
2005	2	2	2
2006	4	4	4
2007	6	6	7
2008	7	7	8
2009	8	8	9
2010	13	12	13
2011	23	22	24
2012	35	34	36
2013	42	40	44
2014	55	53	58
2015	61	59	64
2016	67	64	69
2017	69	66	72
2018	70	67	73
2019	71	68	74
2020	72	68	75
2021	73	69	76
2022	74	70	77
2023	75	70	78
2024	76	71	79
2025	77	72	80

Green highlighted rows show projection in trend (a linear projection from trend in 2018-2020)

Section 2: Scenario setters

Scenario	Name	Affiliation
China NTP	Lixia Wang	Director, National Center for Tuberculosis Control and Prevention, Chinese Center for Disease Control and Prevention, Beijing, China
	Daniel Chin	Bill and Melinda Gates Foundation, China Office, Beijing, China
India NTP	Vineet K Chadha	Head, Epidemiology and Research Division, National Tuberculosis Institute, Bangalore, India.
	Kiran Rade	National Consultant TB Epidemiologist, World Health Organization, Country Office for India, New Delhi, India (Central TB Division, WHO-RNTCP Technical Assistance Project,)
	Puneet Dewan	Senior Programme Officer, TB The Bill & Melinda Gates Foundation, New Delhi, India
RSA NTP	Yogan Pillay	Deputy Director General of HIV, TB and Women and Child Health Department, National Department of Health, Pretoria, South Africa
	L David Mametja	Chief Director South Africa NTP, National Department of Health, Pretoria, South Africa
	Michael E Kimerling	Bill and Melinda Gates foundation (currently KNCV Tuberculosis Foundation, The Hague)
Advocacy scenarios	Sahu Suvanand	Deputy Executive Secretary, Stop TB Partnership, Geneva, Switzerland
	Colleen Daniels	Stop TB Partnership, Geneva, Switzerland
	Lucica Ditiu	Executive Secretary, Stop TB Partnership, Geneva, Switzerland

Appendix 3: INTERVENTION SCENARIOS

A. INTERVENTION SCENARIOS

Six intervention scenarios were defined which aimed to capture the main pathways currently at our disposal to reduce the TB burden (see main paper, Table 2). To enable consistent implementation across different models, we linked each intervention scenario to changes in a set of model parameters that captured the epidemiological impact.

B. PURPOSE OF THIS SECTION

The purpose of this section is to summarise the information modelling groups needed to implement the intervention scenarios consistently, in addition to Table 2 in the main paper, and provide information on implementation approach taken by each model dependent on their structure.

For each intervention scenario, we provided first an overall description together with a table outlining the specific activities included as defined by the scenario setters. Secondly, we provide the guidance on how the intervention should be implemented, given different potential model structures as well as the minimum model functionality required for certain intervention scenarios (e.g. intervention scenarios involving changes to the MDR care pathway would require a model to include resistance-specific strata). Finally a table explicitly summarises key structural approaches for each model what key structure was present, and therefore which approach applied to them.

Intervention scenario 1: Increase access to high quality care

Summary description

This intervention scenario seeks to represent an increase in access and uptake of higher quality TB services. It was divided in two parts:

> Scenario 1.a: an increase proportion of the population with access to health care (from no access to care to access to care).
> Scenario 1.b: an increase in the proportion of TB cases accessing high quality TB care (from accessing low quality care to accessing high quality care)

Conceptually, this is thought of as dividing the population into three subgroups: 1) those with no access to care; 2) those with access to low quality services; and 3) those with access to high quality services. The baseline estimate of the proportion of the population not accessing care was elicited as "percentage of TB case population who will never access care, <u>due to structural barriers</u>". These TB cases will either die or self cure.

The quality of TB services was assumed to potentially <u>affect the diagnosis as well as treatment</u> of TB cases accessing TB services. In <u>China and India</u>, both diagnosis and treatment outcomes were assume to be affected. For <u>South Africa</u>, scenario 1b (increase in the proportion of TB cases accessing high quality TB care) is captured as the implementation of intensified case finding at primary healthcare level (i.e. a policy to screen all PHC clinic attendees for TB symptoms), which <u>only affects diagnosis</u>.

Table 1.1: Activities and supporting evidence of effect

	Activities and coverage	Direct effect description and size	Reference for effect size
CHINA			
**1a**	Removal of financial barriers. Government to pay for TB diagnosis and drugs in both hospital and CDC sector, and compensates patients for incurred (direct medical) costs.<br><br>Coverage of intervention (of all those with active TB): increase to 96.25% from a baseline of 95%.	Effect: Extent to which access is increased per person covered<br>Effect size: 100%, i.e. all those who have access to the government scheme access care.	Assumption
1b	All TB care will be provided by designated hospitals and CDC (shifted from general health centres).<br>All technologies and approaches remain the same as currently in high quality care settings.<br><br>Coverage of intervention (of all those with active TB with access): 95%	Effect: Extent to which people move from low to high quality care<br>Effect size: 95%	Assumption
INDIA			
**1a**	Removal of financial and temporal (longer service hours) barriers. Government to pay a subsidy to private sector to ensure TB diagnosis and treatment is free in this setting.<br><br>Expansion in the number of microscopy centres accessible (double in public sector).<br><br>Coverage of intervention (of all those with active TB): 95.25%	Effect: Extent to which access is increased per person covered<br>Effect size: 100%	Assumption
**1b**	This scenario includes the same subsidy as in intervention scenario 1a, but in this case, the investment brings about an increase in the access to high quality care<br><br>Coverage (of all those with active TB with access): 90%,	Effect: Extent to which people move from low to high quality care<br>Effect size: 100%	(1)

SOUTH AFRICA			
**1a**	Removal of geographical barriers through outreach clinics<br><br>Coverage (of those with active TB): 100%	Effect: Extent to which access is increased per person covered<br>Effect size: 100%	Assumption
**1b**	Intensive case finding at PHC level: All patients attending PHC services will be screened at clinic entrance (i.e. verbal symptom screening). The number of screenings done ensures that 100% of unique individuals attending PHC services are screened every year (e.g. Some attendees might be screened several times in one year).<br><br>Coverage (of those with access): 100%	Effect: increase in rate of diagnosis of TB cases<br>Effect size: see below	(2, 3)

Implementation guidance for intervention scenario

When a model has specific structure differentiating health service sectors on the basis of quality of TB services, TB cases should be redistributed across the different types of care. If a model only has two strata of types of care (e.g. low and high quality), they would be expected to calculate a weighted average for the 'low quality services' (see below).

When model does not have specific structure differentiating health service sectors on the basis of quality of TB service, a **weighted average** for each of the relevant TB services parameters should be calculated, based on the proportion of cases in each sector. TB services parameters include time to diagnosis/diagnostic rate, linkage to care and treatment outcome.

[China] Diagnostic rate ratio is expressed as the relative rate of diagnosis in the HQ sector compared to the LQ sector = 1-4 (assumption). [India] Initial default (private) = Initial default (public); Treatment success (private) = 35-40% DS, 10% for MDR

[South Africa] (intervention scenario 1b):
We calculated a change in rate of diagnosis based on the number of individuals covered by ICF at PHC clinics. The number of unique individuals screened during one year assumes a total of 20mln eligible unique visitors to primary health care facilities, and a 25% refusal rate. Based on Claassens et al, 63% (2) of those screened will enter the diagnostic pathway, and 5% will be diagnosed with TB. Based on the current diagnostic algorithm (Xpert), we will translate the total number of extra diagnoses into a 100% increase in the rate of diagnosis for all patients HIV negative, HIV positive (receiving or not ART). Models without a diagnostic rate parameter should approximate this impact. Note: all patients diagnosed through ICF are assumed to enter the same TB care pathway as those diagnosed through other means.

Table 1.2 Modelling approach

Model	Explicit structure in model (if no – weighted average approach used)	
	**No access to care**	**Different Quality of Care sectors**
TIME	No	No
Hopkins	No	Yes
Harvard	Yes	Yes
STAMP	No	Yes
IDM	Yes	Yes
IRD	Not modelled	
SIPTM	Not modelled	
ICPHFI	Yes	Yes
UGA	No	Yes
AuTuMN	Yes	Yes
NTU	Yes	Yes

Intervention scenario 2: Diagnosis of disease and MDR

Summary description

This intervention sought to represent the impact of replacing smear as the first laboratory test by a molecular test (e.g. Xpert or similar). This would only affect TB patients accessing care in the 'high quality' sector. This intervention scenario has been modelled for India and China only. South Africa has added Xpert to the current standard of care.

Two key model parameters are affected

a) diagnosis rate of smear negative DS pulmonary TB
b) diagnosis rate of MDR TB.

Summary of evidence for epidemiological effect

- Test characteristics – assuming performance is identical to Xpert (4)
    - As initial test for pulmonary TB, sensitivity is 89% (85% to 92%) and specificity is 99% (98% to 99%)
        - Among smear-positive patients, sensitivity is 98% (97% to 99%)
        - Among smear-negative patients, sensitivity is 68% (59% to 74%)
    - As a test for detection of rifampicin resistance, sensitivity is 95% (90% to 97%) and specificity is 98% (97% to 99%).
- Xpert has the potential to increase diagnosis of smear-negative <u>pulmonary</u> TB compared to smear microscopy
- Xpert has the potential to increase diagnosis of rifampicin resistance (as proxy for MDR diagnosis)
- From operational trials in South Africa, there is evidence to suggest Xpert improves the sensitivity of the diagnostic algorithm. However, no evidence was found of impact on higher level outcomes, such as number of cases started on treatment or on mortality, most likely due to high levels of empiric treatment, as shown in the TB NEAT (5) and XTEND (3) studies

For this exercise, we assumed the effect on additional diagnoses would be limited to smear negative pulmonary TB cases only. As an approximation of the impact of Xpert on smear negative diagnosis, in the context of empirical treatment, results from Theron et al (Figure 3, (5)) have been used. It can be estimated that the use of Xpert improves the sensitivity of TB diagnosis in smear negative patients by 23% compared to microscopy (row 3, from 67% to 83%). The calculation of this value can be found in table 2.1.

Table 2.1: Calculations for reduction in false positive and false negative rate with a diagnostic algorithm including Xpert

	Smear Alg	Xpert Alg	Notes
Algorithm sensitivity (all TB)	0.84	0.91	(5)
Fraction of TB smear-positive/ xpert-positive	0.49	0.82	(5)
Algorithm sensitivity (smear-negative TB)	0.67	0.83	Calculation, assumes perfect sensitivity in smear-positive patients
Algorithm false-negative rate (smear-negative TB)	0.33	0.17	Calculation
**Reduction in false negative rate with Xpert**	**---**	**48%**	Calculation
Algorithm specificity	0.71	0.74	(5)
False positive rate (1-specificity)	0.29	0.26	Calculation
**Reduction in false positive rate with Xpert**	**---**	**8%**	Calculation

Note: the overall impact depends strongly on the baseline level of diagnosis of smear negative TB (e.g. through empirical diagnosis). Sensitivity values among smear negative TB patients of the existing algorithm have been estimated by the NTPs.

Table 2.2: Activities and supporting evidence of effect

	Activities and coverage	Direct effect description and size	Reference for effect size
CHINA AND INDIA			
**2**	Xpert replaces smear where smear is available	The introduction of Xpert has no impact on initial default. The direct effect of the introduction of Xpert on number of new MDR cases started on treatment = coverage of Xpert * sensitivity of Xpert for Rif resistance (0.95) * MDR initial default.<br>In addition, we assumed a reduction of 48% of false negative diagnosis.	(3, 5)

Implementation guidance

a) MDR diagnosis

Depending on the model structure available, the effect of additional MDR diagnostic capacity can be implemented through a change in the delay until accurate diagnosis of TB cases with a dominant MDR strain, or the % of diagnosed TB cases who receive a drug sensitivity test (DST).

If implemented through a change in the delay of diagnosis of MDR TB cases: The impact of Xpert is to reduce the delay of MDR TB diagnosis by the % of additional TB suspects for whom Xpert is part of the initial diagnosis of TB disease. For example, if the coverage goes from 30% to 65%, then the difference between MDR and DS rate of diagnosis should be halved.

If implemented through a change in DST coverage:DST coverage at baseline should reflect the current levels. Following the intervention, increases in DST coverage should be reflected.

If the model has stratification by treatment history, the increase in DST should be distributed as follows: first increase DST for retreatment cases to 100%, then distribute remaining increases to new cases.

[India] Current policy in India includes plans to roll out DST for 100% of retreatment cases by 2019. This is estimated to be ~20% of all TB cases and a separate activity to the Xpert roll-out intervention scenario.

If model does not have retreatment structure: Baseline should include 20% DST in TB case population accessing high quality care, rise from 2015 to 2019
Intervention scenario should increase DST coverage in target population to 44% by 2019

If model has retreatment structure: Baseline should include 100% DST for retreated TB cases, rise from 2015 to 2019.

Intervention scenario should increase DST coverage to 100% of retreated and 22.5% of new cases by 2019

b) Smear negative pulmonary TB

The sensitivity of the diagnostic algorithm for smear negative TB cases should be improved to reflect a reduction of 48% of false negative diagnosis and 8% of false positive diagnosis.

[China] The data on performance of the smear-negative diagnostic algorithm in China are not strong. Models were asked to implement the values of sensitivity = 63% and specificity = 96% (Table 19, p62, Toman's (6)) for this algorithm. In the intervention scenario including Xpert, algorithm sensitivity for smear negative TB = 81% and the specificity = 96.3%

[India] The baseline sensitivity of the diagnostic algorithm for smear negative TB cases = 28% (7) and specificity = 92%. (8) In the intervention scenario including Xpert, sensitivity for smear negative TB = 63%, and specificity = 93%

Table 2.3 Modelling approach

Model	MDR diagnosis through DST coverage
TIME	Yes
Hopkins	Not modelled for South Africa
Harvard	Yes
STAMP	Yes – increase in smear negative diagnosis modelled as weighted average in sensitivity of diagnostic pathway
IDM	Yes
IRD	Not modelled for South Africa
SIPTM	Not modelled for South Africa
ICPHFI	Yes
UGA	Not modelled for South Africa
AuTuMN	Yes
NTU	Yes

Intervention scenario 3: Improve high quality TB services - post diagnosis

Summary description

This intervention seeks to represent improving post-diagnosis TB services. Conceptually, this intervention scenario is restricted to the 'high quality' sector by reducing pre-treatment loss to follow-up and increasing treatment success:

Scenario 3a: Increased linkage into care (reduced pre-treatment loss to follow-up), for both DS and MDR TB
Scenario 3b: Improving treatment success (including reducing on-treatment loss to follow up) for DS TB
Scenario 3c: Improving treatment success (including reducing on-treatment loss to follow up) for MDR TB

Table 3.1: Activities and supporting evidence of effect

	Activities and coverage	Direct effect description and size	Reference for effect size
CHINA			
**3a (DS)**	Removal of financial barriers. Providing free treatment and compensation for direct patient costs (diagnosis related costs only).<br>Coverage of intervention (of DS suspects) is: 100%	Effect: Extent of retention until start treatment per person diagnosed<br>Effect size: 98.5%	(9)

**3a (MDR)**	Removal of financial barriers., compensation for direct patient costs (diagnosis related costs only).<br><br>Coverage of intervention (of MDR-TB suspects) is: 100%	Effect: Extent of retention until start treatment per person diagnosed<br>Effect size: 85%	Assumption
3b	Patient support to improve adherence, and case management strategies.<br>Coverage (of those starting DS treatment) is 100**%**	Effect: Extent of treatment success per person starting treatment<br>Effect size: 8% (from 82% to 90% treatment success)	Assumption
3c	Improvement of case management strategies and patient compensation of direct costs (treatment related costs only)<br><br>Coverage (of those started on MDR treatment) 100%	Effect: Extent of treatment success per person starting treatment<br>Effect: 30% (from 35% to 65% treatment success)	(10)
INDIA			
**3a (DS)**	Patient incentives provided to those providing sputum<br>Coverage (of all suspects) 100%	Effect: Extent of retention until start treatment per person diagnosed<br>Effect size: 95%	(9)
**3a (MDR)**	Linkage to social welfare programmes (including nutritional support)<br><br>Coverage (of MDR-TB suspects) 100%	Effect: Extent of retention until start treatment per person diagnosed<br>Effect size: 95%	Assumption
3b	Patient incentives provided to those on treatment.<br><br>Linkage to social welfare (including nutritional support)<br><br>Coverage (of those started on DS treatment) 100%	Effect: Extent of treatment success per person starting treatment<br>Effect: 10% (from 75% to 85% treatment success)	Assumption

3c	Int 3c:<br>Patient incentives provided to those on treatment<br>Linkage to social welfare (including nutritional support),<br><br>Coverage (of those started on MDR treatment) is 100%	Effect: Extent of treatment success per person starting treatment<br>Effect: 19% (from 48% to 67%)	(11)
SOUTH AFRICA			
**3a (DS)**	Medical officers to improve M&E. M-health and outreach teams to trace patients in the community<br>Coverage (of suspects) 100%	Effect: Extent of retention until start treatment per person diagnosed<br>Effect size: 95%	(9)
**3a (MDR)**	Medical officers to improve M&E. M-health and outreach teams to trace patients in the community<br>Coverage (of MDR-TB suspects) 100%	Effect: Extent of retention until start treatment per person diagnosed<br>Effect size: 95%	Assumption
3b	Patient adherence counselling and psychosocial support, tracing<br>M&E boosting<br><br>Coverage is 100% of cases started on DS treatment	Effect: Extent of treatment success per person starting treatment<br>Effect size: 9% (from 80% to 91% treatment success)	Assumption
3c	Mhealth, adherence counselling, psychosocial support, tracing<br><br>M&E boosting. New officers being appointed, decentralisation of Electronic register<br>Coverage (of those starting MDR-TB treatment) is 100%	Effect: Extent of treatment success per person starting treatment<br>Effect size: 15% (from 52% to 67% treatment success)	(11)

Implementation guidance

[South Africa] The improvement in ART access should reduce TB treatment mortality under all scenarios. For those models which do not have functionality to deal with this automatically (i.e. contribution of HIV mortality to total treatment mortality and success rates modelled separately), this effect can be implemented via an additional linear increase in success rates (3% for DS-TB regimens, 5% for MDR-TB regimens), introduced between 2015 and 2025.

If a model explicitly captured death during treatment, and this is dependent on ART status: to avoid double counting effect of ART, you should reduce the target for 3b and 3c to take out the ART induced reduction in mortality (i.e. from 85 to **82% for DS-TB regimens**, from 67 to **62% for MDR -B regimens**). The effect of ART should be captured automatically by the background scale-up of ART.

If your model **does not** explicitly capture death during treatment, or it is not dependent on ART status: for those models which do not distinguish mortality as a separate component of treatment success, an additional step is required to allow for the improvements in TB treatment outcomes that will occur as a result of ART scale-up. The reasoning is that improved access to ART has a direct effect on Rx success through reducing on-treatment mortality.

Table 3.2 Modelling approach

Model	Parameters for Initial default and treatment success (by MDR)
TIME	Yes
Hopkins	Yes
Harvard	Yes
STAMP	Initial default modelled as default in first month of treatment
IDM	Yes
IRD	Not modelled
SIPTM	Not modelled
ICPHFI	Yes
UGA	Yes
AuTuMN	Initial defaulters in explicit compartment. Time spent in compartment dependent on understanding of local epidemiology
NTU	Yes

Intervention scenario 4: Active case finding (ACF) in general population

Summary description

We defined this intervention scenario as a periodical screening of a proportion of the general population for TB disease. Conceptually, this scenario is an ongoing process of screening that completes a round each year (or half year depending on the frequency of screening). TB cases identified are assumed to enter the high quality care diagnostic pathway as other patients. The ACF campaign was stopped at the end of 2025 (advocates scenarios) and 2020 for India NTP scenario. The screening algorithm was determined by NTPs. We did not explicitly model HIV testing through ACF activities.

The effectiveness (i.e. number of cases detected) will be a direct calculation of % screened * sensitivity of screening algorithm.

Table 4.1: Activities and supporting evidence of effect

	Activities and coverage	Direct effect description and size	Reference for effect size
CHINA			
**4**	Advocacy scenario: The screening algorithm includes an X-ray and Xpert	Sensitivity + Specificity of tests<br>Symptom screen:<br>SENS: 70%<br>SPEC: 61%<br><br>Chest Xray:<br>SENS: 90% after symptom screen, 98% as first test (any sign)<br>SPEC: 56% after symptom screen, 75% as first test (any sign)<br><br>Xpert[1]:<br>SENS: 92%<br>SPEC: 99%	(4, 8)
INDIA			
**4**	NTP scenario: The screening algorithm includes an X-ray followed by Xpert<br><br>Advocacy scenario: The screening algorithm includes an X-ray and Xpert	Symptom screen:<br>SENS: 70%<br>SPEC: 61%<br><br>Chest Xray:<br>SENS: 90% after symptom screen, 98% as first test (any sign)<br>SPEC: 56% after symptom screen, 75% as first test (any sign)<br><br>Xpert:<br>SENS: 92%<br>SPEC: 99%	(4, 8)
SOUTH AFRICA			
**4**	Advocacy scenario: The screening algorithm includes only an Xpert	Xpert:<br>SENS: 92%<br>SPEC: 99%	(4, 8)

Implementation guidance

The calculation of the yield of diagnosed cases for each round: % of total population screened * coverage of that population * prevalence of TB in the general population at that time * sensitivity of total screening algorithm of TB.

The intervention should be implemented as an activity over the course of the year. Xpert is part of diagnostic process, so the same proportion of prevalent DS and MDR TB will be diagnosed. Models should assume random resampling of the prevalent pool.

For simplicity and clarity, we assume these diagnosed cases will experience the same post diagnostic pathway as TB cases diagnosed through passive case finding, e.g. the same % initial default and treatment success.

Table 4.2 Modelling approach

**Model**	**All population groups screened randomly across sub-populations (HIV/ART, Health Care strata)**
TIME	Yes
Hopkins	Yes
Harvard	Yes
STAMP	Yes
IDM	Yes (twice a year)
IRD	Not modelled
SIPTM	Not modelled
ICPHFI	Yes
UGA	Yes
AuTuMN	Yes
NTU	Yes

Intervention scenario 5: ACF followed by treatment of latent TB

Summary description

ACF in general population (as in intervention scenario 4) + preventive therapy for those screened negative for TB disease, but positive for latent TB infection (LTBI).

This intervention scenario seeks to represent the provision of preventive therapy for all individuals (excluding those on ART) diagnosed with latent infection in ACF campaign in Intervention scenario #4.

Table 5.1: Activities and supporting evidence of effect

	Activities and coverage	Direct effect description and size	Reference for effect size
CHINA			
**5**	Intervention scenario 4 screening algorithm followed TST or Quantiferon by for those negative<br><br>If LTBI, then isoniazid and rifapentine administered weekly for 12 weeks	IPT reduces TB incidence in HIV neg (RR = 0.4 (0.31 to 0.52)): Effects in HIV neg observed for 2 years or longer (limited by follow up of trial) and reduces TB deaths in HIV neg but no impact on all cause mortality1<br><br>In high adherence (>80%) group over 2 years of follow-up, RR was 0.2 compared to placebo.<br><br>Combination regimen of isoniazid and rifapentine administered weekly for 12 weeks as effective as 9 months INH but with higher completion rates (82% vs 69%)<br><br>Sensitivity of TST/IGRA for Mtb infection, estimated at around 80% for TST and Quantiferon, 90% for T-Spot.<br><br>Specificity of IGRAs >95%. A conservative choice was made to use the 80% sensitivity.	(12-14)
INDIA			
**5**	Intervention scenario 4 screening algorithm followed TST or Quantiferon by for those negative<br><br>If LTBI, then isoniazid and rifapentine administered weekly for 12 weeks	As above	As above
SOUTH AFRICA			
**5**	Intervention scenario 4 screening algorithm followed TST or Quantiferon by for those negative	As above	As above

	If LTBI, then isoniazid and rifapentine administered weekly for 12 weeks		

Implementation guidance

All models modelling this intervention are required to have model structure to deal with patients post preventive therapy, that allows a proportion of those started on preventive therapy to move from a latent state to a state with a lower risk of developing TB (e.g. separate post-PT compartment, or the susceptible compartment) from where they are only vulnerable to re-infection with Mtb.

Of individuals targeted for LTBI treatment, only 80% will receive it, based on the estimated sensitivity of existing tests for LTBI. We assume that before the LTBI screening those with active TB have already been screened out (intervention 4).

We assume only those who complete treatment can experience the protection, i.e. move from the latent compartment to a post IPT state or to susceptible population (depending on assumptions on post-PT immunity. Either option should require post successful-PT individuals to be reinfected before they can develop TB disease.

Note: if models track LTBI with MDR separately, they should model no effect of LTBI Rx on any populations infected with an MDR strain. If models also include the possibility of superinfection with e.g. DS strains, they are free to deal with this at their discretion.

Table 5.2: Model approaches

Model	Post-IPT compartment present
TIME	Yes - no reactivation, immunity as Latent compartment
Hopkins	Yes - no reactivation, immunity as Latent compartment
Harvard	Yes - no reactivation, immunity as Latent compartment
STAMP	No – return to susceptible state, no immunity
IDM	Post-IPT individuals have 35% reduced risk of reinfection
IRD	Not modelled
SIPTM	Not modelled
ICPHFI	?
UGA	Yes - no reactivation, immunity as Latent compartment
AuTuMN	Yes - no reactivation, immunity as Latent compartment (50%)
NTU	No – return to susceptible state, no immunity

Intervention scenario 6: Continuous IPT for ART receiving population

Summary description

This intervention scenario covers provision of continuous IPT to all individuals receiving ART, regardless of TST status. It included a screening component for active TB that consists of Xpert (100% sensitive in smear-positives, ~60% in smear-negatives, and specificity of ~98%, once for all individuals on ART at the start of the intervention, and once for all individuals as they start ART in subsequent years.

To account for the fact that repeat screenings may be required as individuals drop out and return to ART care, we will assume additionally that 5% of the population on ART is screened per year for active TB (note, this 5% value will not apply to models that explicitly model ART drop-out and re-initiation).

IPT is assumed to reduce the risk of active TB, through any mechanism, by 35%. This intervention will nonetheless achieve high coverage given the projected high coverage of ART in South Africa. We make the simplifying assumption here that IPT does not protect against infection, only progression to disease from new or latent infection.

Table 6.1: Activities and supporting evidence of effect

	**Activities and coverage**	**Direct effect description and size**	**Reference for effect size**
SOUTH AFRICA			
**6**	Xpert used for screening active TB<br><br>No follow on clinical diagnosis or additional tests used for Xpert negatives	<u>Effect of IPT for PLWH on ART</u><br>During therapy (in trial of 12m IPT): 37% additional reduction in risk of TB disease during study. No evidence for difference by TST status. (15)<br><br><u>Adherence IPT in combination with ART:</u><br>In the IPT+ART study, lost to follow-up was 11%. This included those who defaulted on the study drug for >3 months. (15)<br><br>Pre IPT screening: here a single Xpert test will be used to screen all those about to start IPT. In HIV positive individuals, Xpert has an overall sensitivity of 79% Specificity is estimated at 98%. (4)	(15) (4)

Implementation guidance

Effect of pre IPT screening:

If a compartment exists where HIV positive individuals on IPT sit, the flow of patients initiating IPT should be adjusted so the correct % of all patients receiving ART is in the IPT compartment, which should include the 5% annual turnover of patients dropping out and re-entering through re-screening. For clarity, we are assuming an equal % on IPT across all CD4 strata within those receiving ART (if these exist).

To reflect the pre-IPT screening for active TB, the following proportion of HIV positive active TB cases should be diagnosed in each time step: PrevTB = prevalence of TB in HIV positive population at time (t), SensTB = Sensitivity of Xpert in HIV pos (79%), SpecTB=specificity of Xpert (98%).

N screened = No. starting IPT / [(1-PrevTB)*SpecTB + PrevTB*(1-SensTB)]

N TB cases diagnosed = N starting IPT * [(1-PrevTB)*(1-SpecTB) + PrevTB*SensTB] / [(1-PrevTB)*SpecTB + PrevTB*(1-SensTB)]

As an approximation, it should give a reasonable estimate the effect of finding TB cases due to screening.

Effect of IPT

It is assumed models will apply a level of protection of progression to disease to individuals in this compartment. For this exercise, protection should apply to disease following recent infection as well as reactivation from existing latent infections.

Table 6.2: Model approaches

Model	How reach coverage level?
TIME	No 'On preventive therapy class', calculation of weighted average of progression based on proportion receiving IPT with ART
Hopkins	IPT initiation rate among ART-receiving population adjusted to reach required coverage
Harvard	IPT initiation rate among ART-receiving population adjusted to reach required coverage
STAMP	Not modelled
IDM	IPT initiation rate among ART-receiving population adjusted to reach required coverage
IRD	No 'On preventive therapy class', calculation of weighted average of progression based on proportion receiving IPT with ART
SIPTM	Preventive therapy class present
ICPHFI	Not modelled
UGA	IPT initiation rate among ART-receiving population adjusted to reach required coverage
AuTuMN	No 'On preventive therapy class', calculation of weighted average of progression based on proportion receiving IPT with ART
NTU	Not modelled

Intervention scenario 7: Combination of 1-6

Summary description

This is the combination of intervention scenarios 1-6 scaled up simultaneously. As currently set up, all interventions scenarios act on different parts of the TB Care cascade (Intervention 1-4) or natural history (intervention 5+6). While there are likely to be many potential interactions between these scenarios that could be considered, there is little to no evidence to guide this. It is assumed that there is no synergy between intervention effects.

Implementation guidance

For the combination of interventions, only models that have provided results for all interventions for that country can participate.

The combination intervention scenario should therefore reflect all the parameter changes that apply to that country. E.g. the increase in the proportion of the TB case population accessing high quality TB services (int 1) happens at the same time while linkage to care treatment success in that sector improve (int 2) and the rate of MDR diagnosis increases, as does the relative detection of smear negative pulmonary TB (int 3). During these changes, ACF is applied to the current prevalent TB disease pool during those simultaneous changes (int 4), and preventive therapy is applied to a proportion of the HIV negative latent disease pool (int 5). If continuous IPT (int 6) is modelled, this intervention happens simultaneously.

Note: all models implemented the combination intervention as a simultaneous change in the parameters affected by each intervention scenario, to the same extent.

References

1. Central TB Division DGoHS, Ministry of Health & Family Welfare,. National Strategic Plan for Tuberculosis Control 2012-17. New Delhi: 2012.
2. Claassens MM, van Schalkwyk C, du Toit E, Roest E, Lombard CJ, Enarson DA, et al. Tuberculosis in healthcare workers and infection control measures at primary healthcare facilities in South Africa. PLoS One. 2013;8(10):e76272.
3. Churchyard GJ, Stevens WS, Mametja LD, McCarthy KM, Chihota V, Nicol MP, et al. Xpert MTB/RIF versus sputum microscopy as the initial diagnostic test for tuberculosis: a cluster-randomised trial embedded in South African roll-out of Xpert MTB/RIF. The Lancet Global health. 2015;3(8):e450-7.
4. Steingart KR, Schiller I, Horne DJ, Pai M, Boehme CC, Dendukuri N. Xpert(R) MTB/RIF assay for pulmonary tuberculosis and rifampicin resistance in adults. Cochrane Database Syst Rev. 2014;1:CD009593.
5. Theron G, Zijenah L, Chanda D, Clowes P, Rachow A, Lesosky M, et al. Feasibility, accuracy, and clinical effect of point-of-care Xpert MTB/RIF testing for tuberculosis in primary-care settings in Africa: a multicentre, randomised, controlled trial. Lancet. 2014;383(9915):424-35.
6. Toman K. Toman's tuberculosis case detection, treatment, and monitoring: questions and answers - 2nd edition (available at http://apps.who.int/iris/bitstream/10665/42701/1/9241546034.pdf). Frieden T, editor. Geneva: World Health Organisation; 2004.

7. Weyer K, Mirzayev F, Migliori GB, Van Gemert W, D'Ambrosio L, Zignol M, et al. Rapid molecular TB diagnosis: evidence, policy making and global implementation of Xpert MTB/RIF. Eur Respir J. 2013;42(1):252-71.
8. WHO. Systematic screening for active tuberculosis: principles and recommendations. Geneva: World Health Organisation, 2013.
9. Lutge E, Lewin S, Volmink J, Friedman I, Lombard C. Economic support to improve tuberculosis treatment outcomes in South Africa: a pragmatic cluster-randomized controlled trial. Trials. 2013;14:154.
10. Li R, Ruan Y, Sun Q, Wang X, Chen M, Zhang H, et al. Effect of a comprehensive programme to provide universal access to care for sputum-smear-positive multidrug-resistant tuberculosis in China: a before-and-after study. The Lancet Global health. 2015;3(4):e217-28.
11. Gelmanova IY, Taran DV, Mishustin SP, Golubkov AA, Solovyova AV, Keshavjee S. 'Sputnik': a programmatic approach to improve tuberculosis treatment adherence and outcome among defaulters. Int J Tuberc Lung Dis. 2011;15(10):1373-9.
12. Smieja MJ, Marchetti CA, Cook DJ, Smaill FM. Isoniazid for preventing tuberculosis in non-HIV infected persons. Cochrane Database Syst Rev. 2000(2):CD001363.
13. Pai M, Denkinger CM, Kik SV, Rangaka MX, Zwerling A, Oxlade O, et al. Gamma interferon release assays for detection of Mycobacterium tuberculosis infection. Clinical microbiology reviews. 2014;27(1):3-20.
14. Jereb JA, Goldberg SV, Powell K, Villarino ME, Lobue P. Recommendations for use of an isoniazid-rifapentine regimen with direct observation to treat latent Mycobacterium tuberculosis infection. Morbidity and Mortality Weekly Report 2011(1545-861X (Electronic)).
15. Rangaka MX, Wilkinson RJ, Boulle A, Glynn JR, Fielding K, van Cutsem G, et al. Isoniazid plus antiretroviral therapy to prevent tuberculosis: a randomised double-blind, placebo-controlled trial. Lancet. 2014;384(9944):682-90.

Section 4 - Additional figures

A1a: Additional calibration targets for China (prevalence of adult SSpos TB in 2000 and 2010)

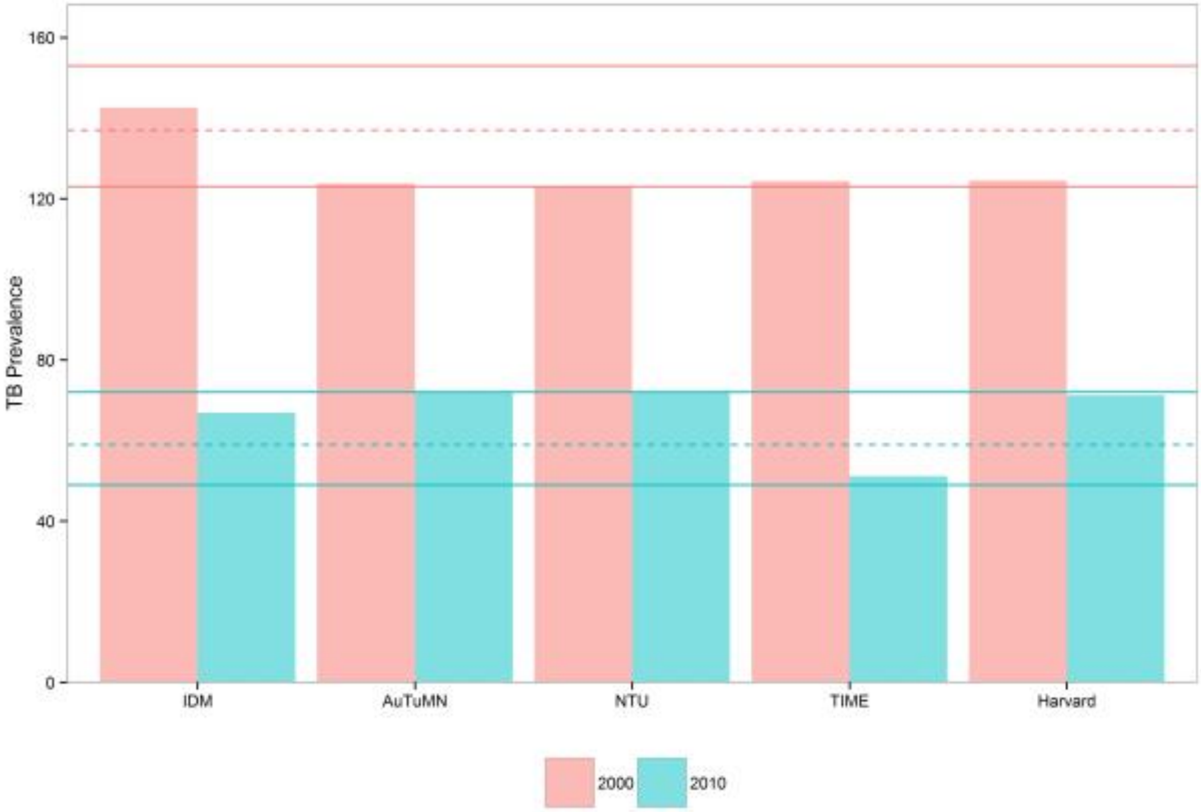

Figure A1a shows adult smear positive TB prevalence in China as estimated by models in 2000 (pink bars) and 2010 (blue bars). Horizontal lines show observed data mid-point (dotted line) and 95% range (solid line).

A1b: Additional calibration targets for South Africa (annual decline in 2012)

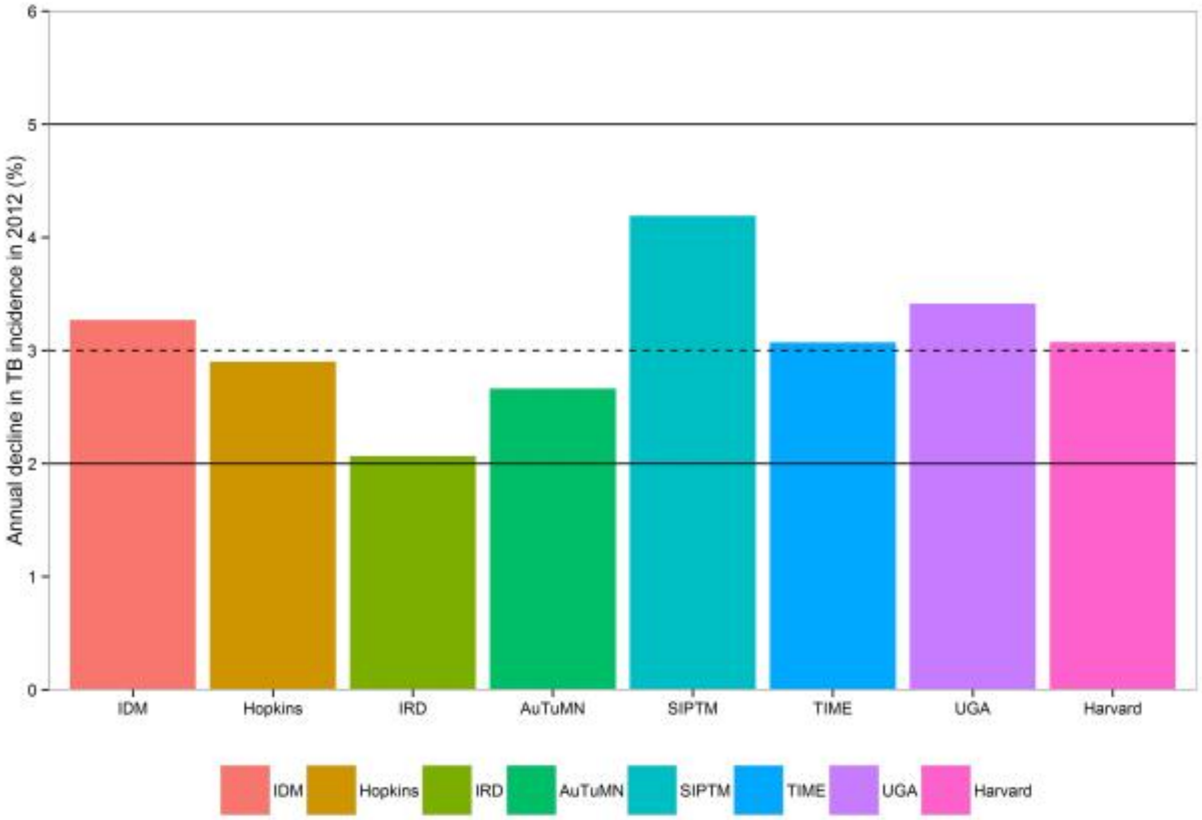

Figure A1b shows the annual decline in incidence as estimated by models in 2012 (bars). Horizontal lines show calibration range between 2 and 5% (solid line) and point estimate of 3% (dotted line).

A2: Impact of interventions on mortality for NTP and Advocacy scenarios

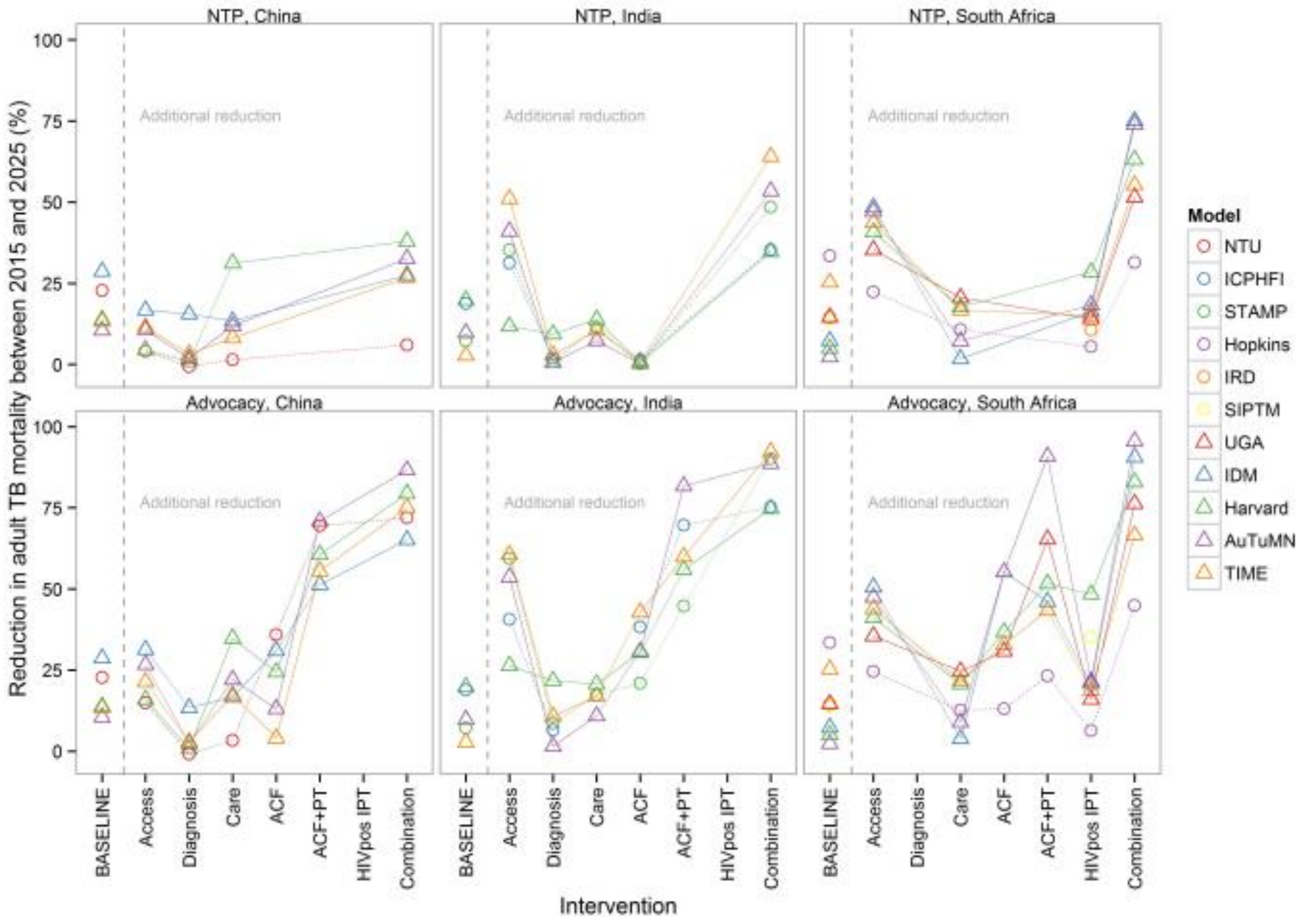

Supplement: Supplementary appendix [file mmc1.pdf]
